# Multiple Myeloma as a Potential Cause of Low Complements in Patients With Acute Kidney Injury

**DOI:** 10.7759/cureus.59056

**Published:** 2024-04-26

**Authors:** Kimberly Q Nguyen, Alexander Ting, Lorraine I Chong Tai, Philip Helderlein, Parham Eftekhari

**Affiliations:** 1 Internal Medicine, Broward Health Medical Center, Fort Lauderdale, USA; 2 Dr. Kiran C. Patel College of Osteopathic Medicine, Nova Southeastern University, Fort Lauderdale, USA; 3 Nephrology, Broward Health, Fort Lauderdale, USA

**Keywords:** c3 glomerulopathy, myeloma kidney, complement c3, hypocomplementemia, multiple myeloma

## Abstract

Multiple myeloma (MM) is a plasma cell malignancy belonging to the class of monoclonal gammopathies that leads to end-organ damage myeloma events that encompass anemia, the presence of lytic bone lesions, hypercalcemia, and renal insufficiency. However, there are very few reported cases of patients with low complements in the context of MM and renal failure. Traditionally, low complements in glomerular disease are associated with conditions such as membranoproliferative glomerulonephritis, cryoglobulinemia, systemic lupus erythematous, and post-infectious glomerulonephritis. Despite its rarity, physicians should maintain a high degree of suspicion and consider MM as a potential cause of low complements in patients with renal injury. In this case report, we present a patient with a history of MM associated with acute kidney injury with hypocomplementemia, an atypical presentation of myeloma in MM.

## Introduction

Multiple myeloma (MM) is a plasma cell malignancy belonging to the class of monoclonal gammopathies that leads to end-organ damage myeloma events that encompass anemia, the presence of lytic bone lesions, hypercalcemia, and renal insufficiency [1]. End-organ damage events are referred to as myeloma-defining events and encompass anemia, the presence of lytic bone lesions, hypercalcemia, and renal insufficiency. Diagnostic markers include a clonal bone marrow plasma cell percentage greater than or equal to 60%, serum free light-chain ratio ≥100, or more than one focal lesion on MRI [2]. Kidney injury secondary to MM can result from a variety of factors, including cast nephropathy, monoclonal immunoglobulin (Ig) deposition disease, and amyloid light-chain amyloidosis. Less common mechanisms of injury encompass cryoglobulinemia, minimal change disease, thrombotic microangiopathy, and glomerulonephritis [3,4]. At a certain level, light chains can cause renal impairment due to abnormal deposition of complements [5]. The abnormal deposition of complements in the glomeruli can cause an abnormal activation of the alternative pathway of the complement system [6]. However, the association of hypocomplementemia with MM remains poorly understood besides cases of cryoglobulinemia. In this case report, we present a patient with a history of MM associated with acute kidney injury (AKI) with hypocomplementemia.

## Case presentation

**Figure 1:**
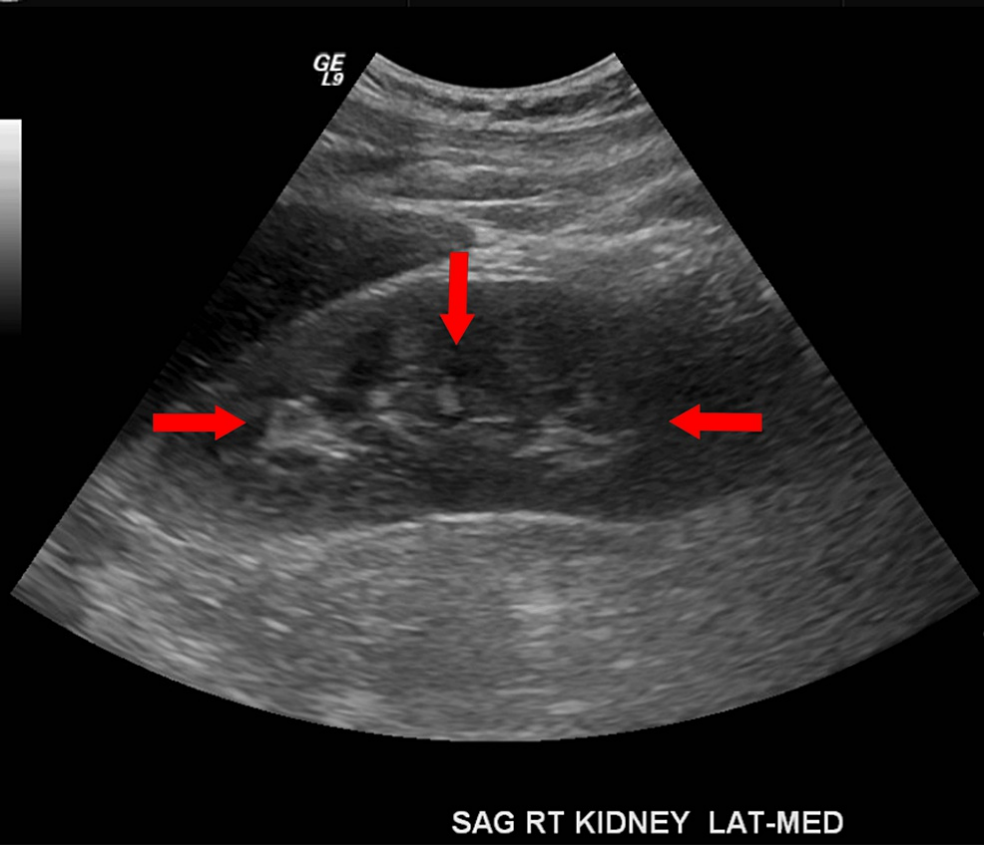
Renal ultrasound showing an echogenic kidney consistent with chronic medical renal disease (as indicated by arrows).

A 60-year-old male with a history of MM, currently in remission for the past three years, hypertension, asthma, and osteoarthritis initially presented to the emergency department due to an altered mental status that had persisted for one week. The physical examination was largely noncontributory, except for the noted confusion. The neurological examination was otherwise unremarkable. Vital signs on admission revealed a tachycardic patient with a heart rate of 126 beats per minute, a blood pressure of 166/100 mmHg, a respiratory rate of 20 respirations per minute, and an oxygen saturation of 95% on room air. Lab results showed a sodium level of 122 mmol/L, a calcium level of 12.2 mg/dL (corrected calcium level of 13.7 mg/dL), a troponin I level of 0.31, low complements, and a hemoglobin level of 7.7 g/dL (Table 1). Creatinine was 2.3 mg/dL, blood urea nitrogen was 25 mg/dL, and estimated glomerular filtration rate was 38 mL/minute/1.73m². A workup of hypocomplementemia revealed antinuclear antibodies, antineutrophilic cytoplasmic antibodies, anti-myeloperoxidase antibodies, antistreptolysin O, cryoglobulinemia, and anti-proteinase 3 antibodies were negative. The initial urinalysis revealed moderate hematuria consistent with acute nephritic syndrome, with 30 mg/dL of protein in the urine, six red blood cells (RBCs)/high-power field, three hyaline casts/low-power field, and RBC casts. The electrocardiogram was unremarkable, and the chest X-ray revealed stable interstitial infiltrates compared to a prior examination in 2017. Renal ultrasound showed an echogenic kidney consistent with chronic medical renal disease (Figure 1). The patient was transfused with two units of RBCs and received a bolus of normal saline before being admitted to the medicine service.

**Table 1:** Laboratory results with reference values range.

Laboratory results	Patient’s laboratory results	Reference range
Serum sodium level	122 mmol/L	135–145 mmol/L
Serum calcium level	12.2 mg/dL	8.5–10.3 mg/dL
Blood urea nitrogen	25 mg/dL	6–24 mg/dL
Creatinine	2.3 mg/dL	0.7–1.3 mg/dL for men
Hemoglobin level	7.7 g/dL	13.8–17.2 g/dL
Troponin level	0.31 ng/mL	0–0.04 ng/mL
Complement 3 level	10 mg/dL	88–201 mg/dL
Complement 4 level	9 mg/dL	13.8–17.2 mg/dL
Kappa/Lambda ratio	71.42	0.26–1.65

Nephrology was consulted for hyponatremia and AKI vs. AKI on chronic kidney disease. The patient’s original diagnosis of MM had been confirmed by a CT-guided bone biopsy of the left iliac crest on 05/09/2017. The biopsy demonstrated trabecular bone marrow with hypercellular packed marrow with a solid, atypical, increased plasma cellular infiltrate, comprising approximately 95%, with kappa restriction. Immunohistochemical stains were positive for CD-138 and kappa and negative for lambda. These morphologic and immunostaining patterns, in conjunction with the patient’s clinical history of multiple lytic bone lesions and IgG kappa monoclonal gammopathy, confirmed the diagnosis of MM. The patient’s last hospital admission in May 2017 showed no evidence of any kidney injury.

The impaired renal function was initially suspected to be due to cast nephropathy, prompting orders for kappa/lambda (K/L) ratio and urine-free light chain measurements. Urine protein and electrolytes were also ordered. The K/L ratio was elevated at 71.42, suggesting an element of kappa light chain cast nephropathy; however, the patient’s C4 and C3 complement levels were low at 9 mg/dL and 10 mg/dL on repeat labs, respectively. Blood cultures were negative, and no infection was suspected (Table 1). As the patient had nephritic pattern disease with low complements, we entertained a diagnosis of membranoproliferative glomerulonephritis (MPGN) and cryoglobulin renal disease. Of note, serum cryoglobulin was undetected and the patient refused renal biopsy. This prompted us to investigate medical literature on the significance of hypocomplementemia with MM and nephritic syndrome. Hypocomplementemia classically has been reported to be due to cryoglobulin and MPGN in MM. Despite not being able to perform a renal biopsy, we felt it was important to highlight medical literature and case reports of low C3 and C4 in MM.

The patient refused renal biopsy and thus was unable to set renal pathology. Ultimately, the patient’s clinical condition improved. Hypercreatinemia 1.9 trended downward and he was deemed stable for discharge to home. He was stable for discharge home with follow-up appointments in nephrology, hematology-oncology, and neurology. As the complement laboratory workup was negative for other potential sources of hypocomplements, we thought this was an interesting case.

## Discussion

MM is a hematological cancer characterized by the clonal proliferation of malignant plasma cells that produce abnormal monoclonal Ig [1]. This condition results in hypercalcemia, osteolytic lesions, anemia, and renal insufficiency, with renal insufficiency having the most significant impact on overall survival among the four defining myeloma events [5,7].

Patients with MM may present with various glomerular and tubular manifestations. The pathogenesis of myeloma renal injury is due to the nephrotoxic effects stemming from the excessive production of monoclonal Igs and free light chains [8]. The most common renal injury in the context of myeloma is cast nephropathy. Less common renal complications in myeloma include various glomerulopathies, such as light-chain deposition disease and amyloid light-chain amyloidosis.

This case study focuses on a 60-year-old male with MM who had been in remission for three years. He was found to have kidney injury and hypocomplementemia and a bone marrow biopsy confirmed MM. Traditionally, low complements in glomerular disease are associated with conditions such as MPGN, cryoglobulinemia, systemic lupus erythematosus (SLE), and post-infectious glomerulonephritis. However, there are very few reported cases of patients with low complements in the context of MM and renal failure [6,9,10]. Serological workup in our patient was negative for cryoglobulins, SLE, and human immunodeficiency virus, and, unfortunately, the patient did not allow us to perform a renal biopsy. Abnormalities of complement activations in myeloma are not well understood besides conditions in MPGN and cryoglobulins. The defect in C3 activation and deposition, known as C3GN, likely plays a role in myeloma-related kidney injury [6,9,10]. The pathophysiology involves light chains binding to the complement regulator region of factor H, leading to abnormal activation of the alternative pathway of the complement system and subsequent abnormal deposition of complement C3 in the glomeruli, resulting in renal damage [6,9,10].

This case illustrates an atypical presentation of myeloma. Despite its rarity, physicians should maintain a high degree of suspicion and consider MM as a potential cause of low complements in patients with renal injury. Therefore, patients with renal injury in the context of MM and low complements should undergo renal biopsy to determine the type of renal pathology. Given the importance of rapidly reducing the excess serum free light chains for recovery of renal function, an aggressive therapeutic approach is justified in patients with myeloma renal injury [5].

## Conclusions

Although the absence of a diagnostic renal biopsy presents a limitation in our ability to conclusively determine the etiology of hypocomplementemia in this patient with renal failure, clinicians should consider the novelty of hypocomplementemia in the setting of MM with AKI, and, if positive, further workup for diagnostic significance with renal biopsy and cryoglobulin levels. We highlight the importance of incorporating complement evaluations into the routine workup for MM-associated renal failure to further investigate causality. Recognizing that the etiology of renal failure associated with MM may extend beyond the common assumption of cast nephropathy, which is the most common manifestation. Clinicians are encouraged to take a proactive approach by challenging the automatic associated with cast nephropathy and broadening the spectrum of potential causative factors. This case report serves as a call to the medical community, emphasizing the need for clinicians to investigate low complements further in MM cases. This signals the need for a comprehensive evaluation and a departure from the conventional assumption of cast nephropathy as the primary manifestation of MM-associated renal failure.
